# A semiparametric cluster detection method — a comprehensive power comparison with Kulldorff's method

**DOI:** 10.1186/1476-072X-8-73

**Published:** 2009-12-31

**Authors:** Shihua Wen, Benjamin Kedem

**Affiliations:** 1 Abbott Laboratories, Abbott Park, IL, USA; 2 Department of Mathematics, University of Maryland, College Park, USA

## Abstract

**Background:**

A semiparametric density ratio method which borrows strength from two or more samples can be applied to moving window of variable size in cluster detection. The method requires neither the prior knowledge of the underlying distribution nor the number of cases before scanning. In this paper, the semiparametric cluster detection procedure is combined with Storey's *q*-value, a type of controlling false discovery rate (FDR) method, to take into account the multiple testing problem induced by the overlapping scanning windows.

**Results:**

It is shown by simulations that for binary data, using Kulldorff's Northeastern benchmark data, the semiparametric method and Kulldorff's method performs similarly well. When the data are not binary, the semiparametric methodology still works in many cases, but Kulldorff's method requires the choices of a correct probability model, namely the correct scan statistic, in order to achieve comparable power as the semiparametric method achieves. Kulldorff's method with an inappropriate probability model may lose power.

**Conclusions:**

The semiparametric method proposed in the paper can achieve good power when detecting localized cluster. The method does not require a specific distributional assumption other than the tilt function. In addition, it is possible to adapt other scan schemes (e.g., elliptic spatial scan, flexible shape scan) to search for clusters as well.

## Background

Scan statistics arise when scanning in time, or space, or both, looking for clusters of certain events or cases. Here the *cluster *can be defined as a certain spatial or temporal subregion where the the probability distribution of an event is different from that in the rest of the region. More generally, a cluster is a subregion where the behavior of an observable is different from the behavior of the observable in the rest of the region. For instance, a subregion comprised of several counties with higher rate of one certain type of cancer than the rate of other counties in the study region defines a cancer cluster [1]. Besides epidemiology, more examples of clusters can be found in many other fields, including criminology, genetics, mining, astronomy, and so on. See Glaz and Balakrishnan (1999), Glaz et al. (2001) [2,3].

As a contemporary research topic, research on scan statistics can be traced back to the 1960's [4]. Since then it came out many scan statistics methods, such as scan statistics with Poisson assumptions [3], scan statistics in purely temporal domain [5], Upper-level set scan [6-8], Flexible shaped scan [9,10], etc. Among them, Kulldorff's spatial scan statistics has been one of the most popular methods and has been applied in many scientific fields for detecting either purely spatial or higher dimensions clusters, e.g. space-time scan [11-15]. However, Kulldorff's method requires assumptions on the underlying distribution (Bernoulli, Poisson, exponential, etc.) of the scan region. If an inappropriate model is chosen, the power of cluster detection may significantly decrease [16]. In addition, the method requires knowledge of the number of events over the region of interest for Monte Carlo hypothesis testing.

Kedem and Wen (2007) [16] proposed a semiparametric cluster detection procedure which requires much less than complete distributional assumptions, and which does not require the number of cases prior to scanning. They also performed a limited power study showing that when data are largely compatible with the Bernoulli or Poisson assumptions, the semiparametric method could be as powerful as the focused test [17] and as Kulldorff's method, but the semiparametric method could achieve higher power when data deviate from the Bernoulli or Poisson assumptions. However, the power study did not involve a scanning stage where the moving window scan the whole study region for cluster candidates. It was assumed that the cluster was already known, and only wanted to compare which method, either Kulldorff's method or the semiparametric one, had higher power in detecting the difference correctly between the cluster region and the background. Hence, the power study was called limited. Although the case study for the North Humberside childhood leukemia data set [11,18] of the paper had the scan stage, the significant level was not adjusted for multiple testing yet.

In this paper, we extended the original semiparametric cluster detection procedure by incorporating Storey's *q*-value method [19,20], a type of false discovery rate (*FDR*) methodology [21], to take into account the multiple testing problem inherent in cluster detection. A comprehensive power study was then conducted using Kulldorff's northeastern benchmark data set [22] for binary power comparison and generated quantized normal data for non-binary power comparison. The results show that for binary data the semiparametric method and Kulldorff's method are quite comparable. However, when data are not binary, the semiparametric method performs well regardless of the probability model whereas Kulldorff's method requires the correct model in order to get a comparably good power. When the chosen model is inappropriate, Kulldorff's method may not achieve good power. This findings are also consistent with the limited power study performed in Kedem and Wen (2007) [16]. The extended semiparametric method was again illustrated by the North Humberside childhood leukemia data set.

## Methods

### Kulldorff's Scan Statistics

**Figure 1:**
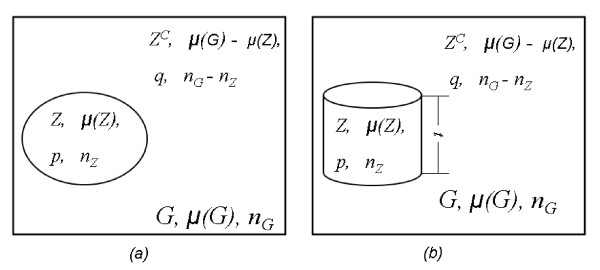
**Notation for Kulldorff's method: (a) two-dimensional purely spatial scan, (b) three-dimensional space-time scan.** (cited from Kedem and Wen, 2007) [16].

Kulldorff's scan statistics method uses a large collection of overlapping scan windows to detect clusters, both the location and the size, and evaluate their significance. See Figure 1 for an illustration. For spatial data, the method first imposes a circular window on a map and let the circle centroid move across the study region. For any given centroid, the radius of the window varies continuously from zero to some upper limit. Usually this upper limit is set to be the radius which covers 50% of the whole study region or population. In this way, the method generates a large set of scan windows ℤ with different centroid and size. Under the null hypothesis of no cluster, the underlying behavior of the data throughout the whole study region is the same. Under the alternative hypothesis, there is at least one scan window for which the underlying behavior inside the window is different as compared with its complement, which means any scan window *Z *(*Z *∈ ℤ) could be a potential cluster. In practice, some data are updated periodically. In such cases, space-time scans are used [13,23]. The scanning procedure of space-time scans is almost identical to the purely spatial scan, except that the scan window becomes a three-dimensional cylinder (see part (b) of Figure 1) instead of two-dimensional circle. Since the statistical formulation of space-time scan is identical to the two-dimensional case, we only describe the 2D purely spatial scan in this paper.

By Kulldorff's scan statistics method, each scan window *Z *is associated with a likelihood ratio test statistic *λ *(*Z*) which can be computed based on the chosen underlying probability model and the observed data. The scan window associated with the maximum *λ *(*Z*) is defined as the primary cluster candidate occurring not by random chance. The maximum likelihood ratio itself is called the Kulldorff's spatial scan statistic, and the null hypothesis is rejected for large value. After the spatial scan statistic and the primary cluster candidate are determined, a Monte Carlo hypothesis procedure or a permutation test procedure is executed to generate the probability distribution of Kulldorff's scan statistic under the null hypothesis of no cluster in the study region, and a *p*-value is obtained [23,24].

For different types of data, Kulldorff proposed different scan statistics based on the corresponding probability models.

#### Bernoulli model

Bernoulli-based scan statistic is used when individual entities have only two states such as an individual person having cancer or not. The part (a) of Figure 1 shows a typical setup of Kulldorff's scan statistics method. The following notation is used.

• *G*: the whole study region

• *Z*: the scan window

• *μ*(*G*): the total number of individual entities (e.g. people) in *G*

• *μ*(*Z*): the number of individual entities in *Z*

• *n*_*G*_: the total number of events in *G*

• *n*_*Z*_: the number of events inside *Z*

• *p*: the rate of events that occurred inside the scan window *Z*

• *q*: the rate of events that occurred outside the scan window *Z*

We test the null hypothesis *H*_0_: *p *= *q *of no cluster. It can be shown that, for an alternative hypothesis that there is a hot spot cluster *p *> *q*, each scan window *Z *invokes a likelihood ratio test statistic as in equation (1):(1)

Where . If we were scanning for cold spots, then "> " would change to "< "; if for either hot or cold spots, then it would be "≠ " (Kulldorff, 1999). After all the *λ *(*Z*) are computed, we determine the primary cluster candidate  from , and reject the null for large values of . A Monte Carlo based *p*-value can be obtained from randomization of the cases across the whole study region given the total number of cases.

#### Poisson model

Poisson-based scan statistic is used for the comparison of the number of cases inside and outside a scan window when searching for clusters. Suppose we have a study region composed of *I *sub-regions. For *i *= 1, 2,..., *I*, assume the number of events *x*_*i *_which occur in an "interval" *μ *(*A*_*i*_) is a Poisson process with intensity rate *p *inside the scan window and *q *outside, respectively. Similarly to the Bernoulli model, the likelihood ratio test statistic for each scan window *Z *for testing *H*_0_: *p *= *q *is shown in equation (2):(2)

where *n*_*Z *_is the observed number of cases inside the scan window *Z*, and *e*_*Z *_is the expected number of cases inside the scan window *Z *under the null hypothesis of no cluster. As before, if we were scanning for a cluster other than hot spot, we simply change the inequality sign as needed. After getting all the *λ *(*Z*)'s, find  and the Monte Carlo based *p*-value. When *p *is small, e.g. a rare disease, the Poisson model gives a close approximation to the Bernoulli model and obtains very similar results [11].

#### Ordinal model

Jung et al. (2006) [15] proposed an ordinal model, which is used when individual entities have *K *> 2 ordinal categories such as the different stages of a kidney disease, stage 1, 2, 3, 4 and 5. A higher category may reflect a more serious cancer stage. Suppose the study region consists of *I *sub-regions and the variable of interest is recorded in *K *categories. Each individual in the analytical sample falls into one category. Let *c*_*ik *_be the number of individuals in location *i *who fall into category *k*, where *i *= 1, 2,..., *I *and *k *= 1, 2,..., *K*. Let *C*_*k *_= Σ_*i *_*c*_*ik *_be the number of observations in category *k *across the study region, and *C *= Σ_*k*_*C*_*k *_= Σ_*k*_Σ_*i *_*c*_*ik *_be the total number of observations in the whole study region.

The null hypothesis of no cluster in this model means *p*_1 _= *q*_1_, ..., *p*_*k *_= *q*_*k*_, where *p*_*k *_and *q*_*k *_are the unknown probabilities that an observation belongs to category *k *inside and outside the scanning window, respectively. To detect subregions with high rates of higher stages as compared with the rest of the area, one possible alternative hypothesis could be . Obviously when *K *= 2, the ordinal model set up reduces to the Bernoulli model. Following similar scan procedures as in Bernoulli and Poisson models, the likelihood ratio test statistic for each scan window is:(3)

where  and  are the MLEs of *p*_*k *_and *q*_*k *_under the alternative hypothesis. A "Pool-Adjacent-Violators" algorithm [25,26] can be applied to compute  and . After all the *λ *(*Z*)'s are obtained, get . A Monte Carlo based *p*-value can be obtained by randomization of the observations across the study region given the total number of observations in each category, *C*_1_, *C*_2_,..., *C*_*K*_.

The above gives a brief summary of Kulldorff's scan statistics under Bernoulli model, Poisson model, and ordinal model, respectively. As for other types of scan statistics in Kulldorff's scan statistics family, see Huang et al. (2007) for the exponential model [14], Kulldorff et al. (2007) for the multivariate scan model [27], Kulldorff et al. (2009) for the normal model [28], and Kulldorff et al. (2006) for elliptic window scans [29].

Kulldorff's method has been applied successfully in many applications. However, it requires assumptions on the underlying distribution (Bernoulli, Poisson, exponential, etc.) of the scan region and the knowledge of the number of events over the region of interest for Monte Carlo hypothesis testing. Moreover, It may take considerable amount of computation time to obtain the Monte Carlo based *p*-value.

### Semiparametric Scan Statistics Method

Kedem and Wen (2007) [16] proposed a semiparametric scan statistics method for cluster detection using a density ratio model studied by Fokianos et al. (2001) and Qin and Zhang (1997) [30,31]. Similar to Kulldorff's scanning procedure, the semiparametric scan statistics method also uses a movable variable-size window to scan the whole study region, but performs for each window a two-sample test. Because the moving window generates a large set of overlapping scan windows, it results in a large number of semiparametric test statistics and their corresponding *p*-values as well. The largest test statistic is defined as the semiparametric scan statistic. To take account of the multiple-testing problem induced by the multiple scanning windows, the original *p*-values are replaced by Storey's *q*-values, a type of controlling false discovery rate (FDR) methodology. Then the primary cluster candidate will be the window (subregion) corresponding to the largest test statistic or equivalently the smallest *q*-value. If this *q*-value is less than a pre-decided level (say 0.05), we claim the located cluster candidate is a true cluster. We describe briefly the semiparametric scan statistics method and the *q*-value FDR methodology in the following subsections.

#### Semiparametric Test Statistics

Using Kulldorff's circular window scan scheme, consider a scan window which separates the study region into two parts. This results in two samples,

where ***x***_1 _is the sample inside the scanning window with sample size *n*_1_, and the ***x***_2 _is the sample outside the scanning window with sample size *n*_2_, *g*_*j*_(*x*) is the probability density function of *x*_*ji*_, *j *= 1, 2, *i *= 1,..., *n*_*j*_. Choosing the sample outside the scanning window as the reference sample and *g*_2_(*x*) as the reference density, it is assumed that the density ratio between the density inside the scanning window and the reference density has an exponential form

Here ***h***(*x*) is a known vector-valued function of *x *which may take on a scalar form such as *x *or a vector-valued form such as (*x*, *x*^2^)', *α*_1 _is a scalar, but ***β***_1 _can be a scalar or vector depending on ***h***(*x*). Clearly, ***β***_1 _= **0 **implies *α*_1 _= 0. Therefore, ***β***_1 _= **0 **implies the null hypothesis of no cluster is accepted since the probability distribution inside and outside the scanning window are equal. See Kedem and Wen (2007)and Fokianos et al. (2001) [16,30] for the general setup for *m *≥ 2 samples.

Following the profiling procedure discussed in Qin and Lawless (1994) [32], Qin and Zhang (1997), and Fokianos et al. (2001), we obtain the log-likelihood:(4)

where *t*_*i *_is *i*th observation in the *combined *sample ***t ***= (***x***_1_, ***x***_2_)', and *ρ*_1 _= *n*_1_/*n*_2_.

It follows from the likelihood (4) that the maximum likelihood estimators  and  from the combined sample are asymptotically normal as *n *→∞,(5)

where **Σ **is a special case of ***S*^-1^*V S*^-1 ^**under *q *= 1. See Appendix 2 for the definition of the matrices ***S ***and ***V ***.

The maximum likelihood estimator of the reference (background) distribution function from the combined sample is given as,(6)

Kedem and Wen (2007) [16] proposed three test statistics for testing the null hypothesis *H*_0_: ***β***_1 _= 0, which means no cluster.

##### *χ*_1 _test statistic

where *ρ*_1 _= *n*_1_/*n*_2 _and *Var *[***h***(*t*)] is the covariance matrix of ***h***(*t*) with respect to the reference distribution. It follows under *H*_0 _that *χ*_1 _is approximately distributed as Chi-square with *v *degrees of freedom, where *v *is the dimension of ***β***_1_, which is the same as the dimension of the function ***h***(*x*). The null hypothesis *H*_0_: ***β***_1 _= 0 is rejected for large values of *χ*_1_. In practice, *Var *[***h***(*t*)] is replaced by its estimator [16,30]. In addition, if the data are binary (0-1), the *χ*_1 _statistic can be simplified to a close form of equation (15) in the Appendix 1.

##### *χ*_2 _test statistic

tests for a general linear hypothesis ***Hθ ***= **c**, where ***θ ***= , *w *is the total number of samples excluding the reference sample. ***H ***is *p' *× [(1 + *v*)*w*)] predetermined matrix of rank *v'*, *v' *< (1 + *v*)*w*, **c **is a vector in , and the variance-covariance matrix **Σ **= ***S*^-1^*V S*^-1^**. It follows under *H*_0 _that *χ*^2 ^is asymptotically distributed as *χ*_2 _with (*v'*) degrees of freedom provided the inverse exists [33], and *H*_0 _is rejected for large values. This test is useful when one wants to borrow information from other sources besides the current study region, although we did not use it in this paper.

##### Likelihood ratio test statistic

Under *H*_0_: ***β***_1 _= 0, *LR *is asymptotically approximately distributed as Chi-square with *v *degrees of freedom, and *H*_0 _is rejected for large values. Recall *v *is the dimension of ***β***_1 _and it depends on the choice of the function ***h***(*x*). Similarly, when the data are 0-1 binary, the likelihood ratio statistic reduces to equation (16) in the Appendix 1.

#### Storey's FDR Method and q-value

Controlling the false discovery rate (FDR) is a less conservative way to handle multiple testing problems. It was first proposed by Benjamini and Hochberg (1995) [21]. Since then, the FDR methodology has been further developed and applied to many fields, especially in genomic research [34-36]. FDR is defined to be the expected proportion of falsely rejected hypotheses (false positives) as in equation (10),(10)

where *V *and *R *are defined in Table 1. From the table it is clear that if *m *= *m*_0_, which means all the null hypotheses are true, then FDR is equivalent to the family-wise error rate (FWER). To see that, recalling FWER is defined as *P*(*V *≥ 1), *m *= *m*_0 _makes *S *= 0, and hence  = 1 for all *R *> 0, and therefore *FDR *= 1 * *P*(*R *> 0) = *P*(*V *≥ 1) = *FWER*. If *m*_0 _<*m*, then *FDR *≤ *FWER*, which means a potential gain in power at the cost of increasing the likelihood of making type I errors [21].

**Table 1:** Classification of *m *hypotheses tests

*Hypothesis*	*# of Accept*	*# of Reject*	*Total*
# of true nulls	*U*	*V*	^*m*^0
# of true alternatives	*T*	*S*	^*m*^1
Total	*W*	*R*	*m*

Storey (2002) and Storey et al. (2004) [19,37] improved the original Benjamini and Hochberg's FDR methodology by estimating the *π*_0 _= *m*_0_/*m*, the proportion of true null hypotheses. In addition, Storey used positive FDR (*pFDR*) as in equation (11) instead of FDR as in equation (10).(11)

In many cases, when *m*, the total number of hypotheses, is large, it easily to have significant ones, which makes *Pr*(*R *> 0) ≈ 1. Thus, pFDR (eq. 11) is close to FDR (eq. 10) in numerical value, but pFDR has some conceptual advantages. A thorough motivation of using pFDR rather than FDR can be found in Storey (2003) [20].

The *q*-value is defined to be the pFDR analogue of the *p*-value. It gives the error measurement with respect to pFDR for each observed test statistic of each particular hypothesis. More precisely, the *q*-value of one particular observed test statistic *T *= *t *from a set of tests can be defined to be(12)

where Γ_*α *_is a nested significance region at level *α *[19]. This means the *q*-value is the minimum pFDR at which the corresponding test may be called significant. In this way, *q*-value simultaneously takes into account the multiple testing problem.

In our semiparametric scan situation, we generate a lot of overlapping scan windows, and each window associates to a hypothesis test and a test statistic, so *m *here is usually large. Because of the large *m*, we adopted the algorithm in Storey et al. (2003) [35] to estimated the *q*-value for each scan window as follows:

1. Obtain the *p*-value for each scan window, and sort

2. Estimate *π*_0 _using a cubic spline function. First, for a range of *λ*, say *λ *= 0, 0.01,...,0.95, calculate

Then let  be the natural cubic spline of . Finally, set the estimate of *π*_0 _to be .

3. Calculate

4. For *i *= *m - *1, *m - *2,...,1, calculate the estimated *q*-value for the *i*th most significant one as

5. Choose the region with the largest test statistic, for example, the largest likelihood ratio test statistic, as the primary cluster candidate, and its *q*-value is *q*(*p*(1)), the smallest *q*-value among all the tests. If *q*(*p*(1)) is less than a pre-decided false discovery rate, say *q*(*p*(1)) < 0.05, we claim there is clustering and the located primary candidate is a true cluster region.

The semiparametric approach has several advantages. First, the reference (or background) distribution, *G*(*x*), and all the parameters such as ***β***_1 _are estimated from the *combined data ****t***, not just from a single sample either inside the window or outside the window. Second, for a properly chosen ***h***(*x*), the above tests are quite powerful. The simulation results indicate that the *χ*_1_-test competes well with the corresponding *F*-test [30]. Moreover, Gagnon (2005) [38] shows that for *m *= 2 the *χ*_1_-test can be more powerful than the common *t*-test for a known ***h***(*x*) but unspecified distributions. Third, in testing equidistribution, other than an assumption regarding the tilt function ***h***(*t*), the semiparametric density ratio method does not require distributional assumptions. Fourth, the semiparametric method can be applied to either continuous or discrete distributions. Fifth, since the asymptotic distributions of the above mentioned test statistics are known, in principle there is no need for the time consuming Monte-Carlo methods to compute the *p*-values. Lastly, Storey's *q*-value method can adjust the original *p*-values, and easily handles the multiple testing problem inherent in cluster detection.

### Data Sets for the Power Comparison

Both Kulldorff's and the semiparametric scan statistics methods are suitable for both binary and non-binary data. So we use both types of data to perform the simulated power study. The following two subsections give a brief description of the binary and the non-binary data sets used in the study.

#### Binary Data Set — Northeastern USA Benchmark Data

For binary data scans, we use Kulldorff's Northeastern USA purely spatial benchmark data [22] which consist of 245 counties in northeastern United States, from Maine, New York, Rhode Island, Pennsylvania, Maryland, Washington DC, among others. Each county is graphically represented by its centroid coordinates. The case data, which is the number of people who have breast cancer, are aggregated to each county centroid with the total number of cases in northeastern states (the whole study region) being fixed. The population data of each county are based on the female population of the 1990 census. The benchmark data set contains two types of data, hot-spot clusters and global clustering data, generated in different ways.

##### Hot-spot clusters

Data are generated by a first-order clustering model, where cases are located independently of each other but the relative risk is different in different geographical areas. In this benchmark data set, the cluster region can be either a single region containing one or more counties, or a collection of multiple regions, where the risk of breast cancer is much higher than in the rest of area. Three types of clusters, rural, urban, and mixed, are generated depending on the location of the cluster. A rural cluster is the type of cluster where a small population relative to a large graphical area, such as the Grand Isle county in north Vermont close to the Canadian border. An urban cluster is the type of cluster which has a large population relative to a small graphical area, such as New York county which includes Manhattan. A mixed cluster is the type of cluster where a big city is surrounded by rural areas, such as Allegheny county in western Pennsylvania where Pittsburgh is. See Kulldorff et al. (2003) [22] for more detail.

##### Global clustering

Data are generated by purely second-order clustering model, where any one particular case is randomly located, but the location of cases are dependent on each other. Thus, under the alternative hypothesis of global clustering, cases are clustered wherever they occur in the region. In this benchmark data set, a certain number of cases are first generated to be randomly located throughout the whole northeastern states. These original cases then generate other new cases close by. If each original case generates one additional case, we call them twins; if two additional cases are generated, we call them triplets. The case generation is based on a global chain *rN*-nearest neighbor rule. The global chain is constructed by a Hamiltonian cycle chain which passes through as many counties as possible exactly once and any two counties next to each other on the chain always border each other graphically. For twins, the additional case is assigned to county *j *if Σ_*k*_*I*(*d*_*ik*_<*d*_*ij*_)*n*_*k*_<*rN *≤ Σ_*k*_*I*(*d*_*ik *_≤ *d*_*ij*_)*n*_*k*_, where *n*_*k *_is the population size of county *k*, *N *= Σ_*k*_*n*_*k *_is the total population size, *r *is some constant in the interval of (0,0.5) with the bigger *r *the broader geographical area to spread the cases, and *d*_*ij *_is the distance in one particular direction along the chain connecting county *i *and county *j*. For triplets, the two new cases are assigned in opposite directions along the chain. Data sets corresponding to different *r *were generated from a probability distribution or be determined in advance. Notice that although the data generation mechanism of the second-order clustering model is very different with the first-order clustering model, the resulting point patterns may look quite similar, and hence indistinguishable.

Both hot-spot and global clustering data sets in this benchmark data set have two separate groups of data with a total of 600 and 6000 simulated cases, respectively. For each group the same null hypothesis of no cluster is used where the relative risk for each county is equal, and the cases as well as their locations are independent of each other. In order to perform power comparison, 100000 random data sets with a total of 600 and 6000 cases were generated under the null hypothesis, respectively. They are used to estimate the critical cut-off point of significance for each group. For the alternative hypothesis of clusters which are called *scenarios *in this paper, 10000 random data sets per scenarios were generated to estimate the power using the previous determined cut-off points.

For each group of fixed total cases (600 or 6000 cases), Kulldorff generated 35 hot-spot clustering scenarios and 26 global clustering scenarios for his power comparison. For instance, a scenario of "rural and urban 600, size 4" means that the total of number of cases in the whole study region is 600 and the study region contains two hot-spot clusters. One cluster is in a rural region including four counties, and the other one is in an urban region including four counties also. A scenario of "global clustering twin 6000, exponential 0.02" means that the total number of cases is 6000 and the cases are generated under the alternative hypothesis of global clustering. The value *r *is not constant but randomly generated from an exponential distribution with mean 0.02 among the data set generated under this scenario.

We did not use all the scenarios in Kulldorff's Northeastern US benchmark data in this paper. Instead, we randomly chose one or two scenarios from each clustering pattern. Finally, nine hot-spot clustering scenarios and six global clustering scenarios were used in our power study for the binary data case. After selecting these scenarios, Kulldorff's method with the Poisson model (Bernoulli model is also appropriate) and the semiparametric method with the tilt function *h*(*x*) = *x *were applied to the same data sets in each scenario to compare the power. Because data are binary, the simplified semiparametric likelihood ratio test statistic (eq. 16) is used in the computation. All the tests are two-sided to detect for either high or low valued clusters.

#### Non-binary Data Set — Ordinal Categorical Data

**Figure 2:**
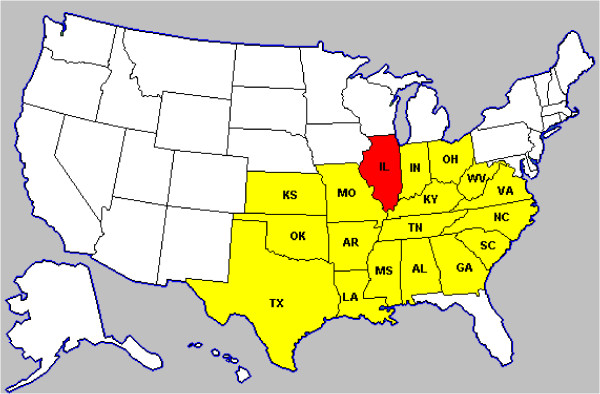
**Map of the US middle west and south states used in the simulation for non-binary data. **The included states are denoted with color and the abbreviation of their state names. The state Illinois with red color is illustrated as one possible cluster region in our simulated data.

For non-binary data scan, we used the simulated ordinal categorical data with one data point corresponding to one observation. The data are aggregated to state level distributed in 18 US states. Most of them are middle west and south states, including Alabama (AL), Arkansas (AR), Texas (TX), Virginia (VA), etc. Each state is graphically represented by its centroid coordinate. Figure 2 shows the map of the states included in our simulation study.

The ordinal categorical data are generated from quantized normal data which truncate the integer part of the original data generated from normal distribution. We refer to quantized normal II and quantized normal III data as in Kedem and Wen (2007) [16]. The quantized normal II data are derived from normal data with the same mean but relatively large variance difference inside and outside the cluster region (inside variance is twice of the outside variance). The quantized normal III data are derived from normal data with both different mean and variance inside and outside the cluster region. To see how Kulldorff's and the semiparametric methods perform when the difference (in mean) between the cluster and the non-cluster region is small, namely a more difficult cluster to be detected, the data set "Quantized Normal III (small)" was generated. We did not generate data under quantized normal I (same variance, different mean) mechanism, because it has shown that as long as the mean difference is reasonably large, Kulldorff's method with even an inappropriate scan statistic still would work [16]. Table 2 lists the mean and variance parameters used in this paper to generate the quantized normal data

**Table 2:** Parameters for Generating the Ordinal Categorical Data from Quantized Normal

*Data Type*	*Inside the cluster*	*Outside the cluster*
Quantized Normal II	*μ *= 13, *σ*^2^= 8	*μ *= 13, *σ*^2^= 4
Quantized Normal III	*μ *= 7.2, *σ*^2^= 13	*μ *= 6, *σ*^2^= 9
Quantized Normal III (small)	*μ *= 6.5, *σ*^2^= 13	*μ *= 6, *σ*^2^= 9

For each type of data, the average sample size within each state is around 130, hence a total number of 130 * 18 = 2340 observations in each generated data set. Cluster patterns included single cluster and multiple clusters for Quantized Normal II, Quantized Normal III, and Quantized Normal III (small) data, respectively. To perform the power comparison within a reasonable time scale, we used 100 runs, namely 100 random data sets, per cluster pattern in our power comparison study. The single clusters means only one state was randomly chosen as the cluster region. By multiple clusters we mean four states were randomly chosen simultaneously as the cluster region, where the four states are not necessarily contiguous. Notice that multiple clusters constitute a stronger clustering pattern which in general is easier to detect.

In our power comparison for non-binary ordinal categorical data, Kulldorff's method chose Poisson model (inappropriate model) and the ordinal model (correct model) using *SaTScan *v7.0.1 http://www.satscan.org/. 999 replications were used to decide the *p*-value under each model. The semiparametric method chose the vector tilt function ***h***(*x*) = (*x*, *x*^2^)'. Observe that it is also appropriate to choose ***h***(*x*) = (*x*, *x*^2^)' for binary data case, because in that case the coefficient of *x*^2 ^term is 0. See Appendix 1 for detail.

## Results

### Power Comparison

We compared the power of Kulldorff's and the semiparametric scan statistics methods in detecting potential clusters. Because there are various cluster patterns, either single or multiple clusters, and each cluster region may contain one or more counties or states, for simplicity, in our power comparison we more focus on detecting the existence rather than the precise delineation of the cluster region. For instance, for a data set with a pattern of multiple cluster regions and multiple counties in each cluster region, we deem the detection successful whenever a significance (i.e., *p*-value or *q*-value less than 0.05) is obtained. We do not strictly require the detected cluster region to be exactly the same as originally simulated. The detected cluster region could fully or partially cover the desired area.

#### Comparison for Binary Data

**Figure 3:**
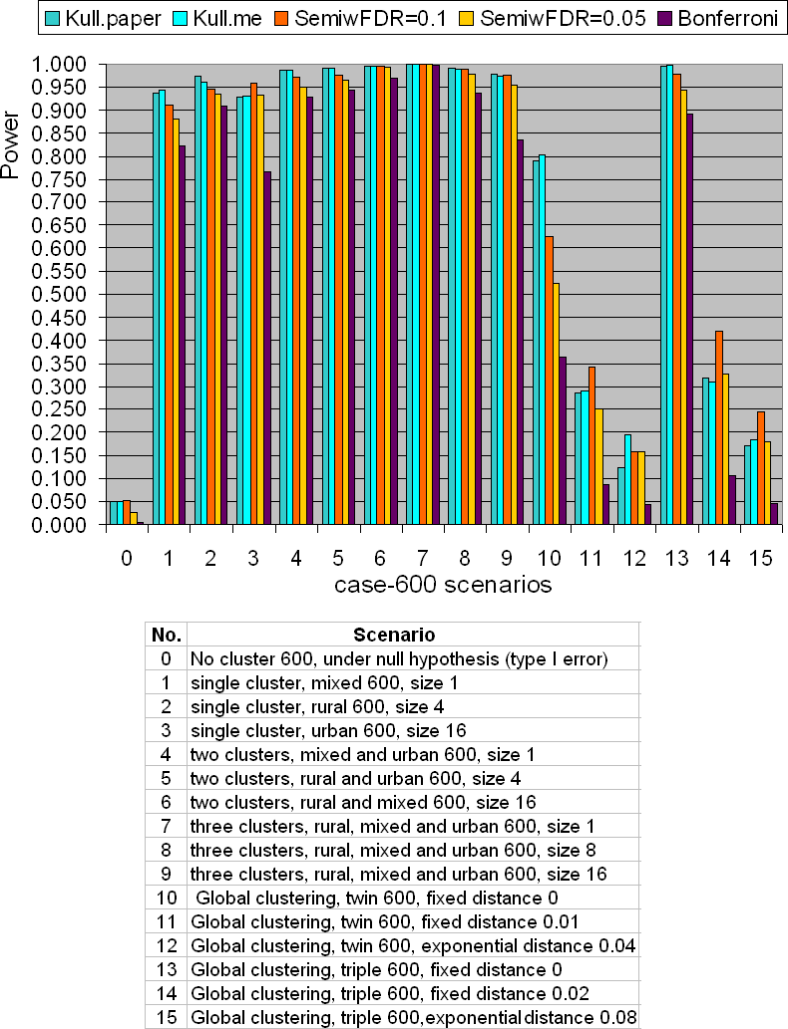
**Power comparison for binary type data between Kulldorff's and the Semiparametric method with the likelihood ratio test using the northeastern US benchmark data with totally 600 simulated cases**.

**Figure 4:**
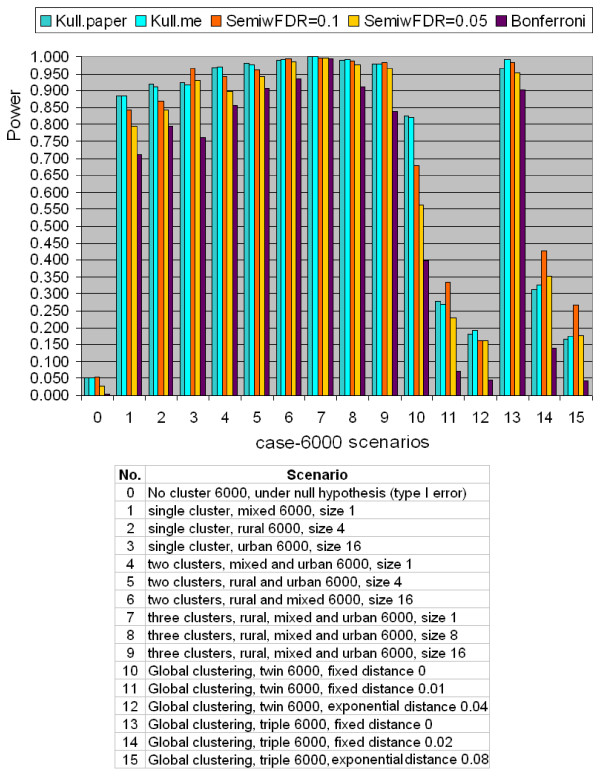
**Power comparison for binary type data between Kulldorff's and the Semiparametric method with the likelihood ratio test using the northeastern US benchmark data with totally 6000 simulated cases**.

The results of power comparison for the binary case-population data using the northeastern US benchmark data set are shown in Figures 3 and 4 for a total number of 600 and 6000 cases, respectively. Kulldorff's method used Poisson model, and the semiparametric method used the likelihood ratio test. All tests are two-sided tests. The radius of the scan window varies continuously from covering one county to covering no more than 50% of the whole study region. In each figure, scenario 0 is under the null hypothesis of no cluster, scenarios 1 to 9 are hot-spot clustering scenarios, and scenarios 10 to 15 are global clustering scenarios. Each scenario contains five quantities. They are "Kull.paper", "Kull.me", "SemiwFDR = 0.1", "SemiwFDR = 0.05", and "Bonferroni". "Kull.paper" is the corresponding power copied from Kulldorff et al. (2003) paper [22]. "Kull.me" is the corresponding power computed by us based on Kulldorff Poisson model. It is shown from Figure 3 and 4 that the column of "Kull.paper" is almost equal to the column of "Kull.me", which confirms the validity of our computation. So in the latter section, we will use "Kulldorff's method" without distinguishing these two power results. "SemiwFDR = 0.1" is the corresponding power computed based on semiparametric method with a *q*-value significance level 0.1. "SemiwFDR = 0.0.5" is the corresponding power based on semiparametric method with a *q*-value significance level 0.05. The smaller the *q*-value significance level is, the harder it is to reject the null hypothesis, hence the lower the power to detect the cluster. "Bonferroni" is the corresponding power computed based on semiparametric method using Bonferroni correction with 5% family-wise error rate, where the original obtained *p*-values are multiplied by the number of tests to obtain Bonferroni corrected *p*-values. If at least one Bonferroni corrected *p*-value is less than 0.05, we claim there is clustering. As we know Bonferroni correction is a popular but conservative approach to handle multiple testing problems, we expect column of "Bonferroni" should have the lowest power among the five.

For scenario 0, both Figures 3 and 4 show that the type I error of Kulldorff's method are all exactly 0.05 for both 600 cases and 6000 cases. This is expected since Kulldorff's method uses a Monte Carlo procedure to derive the cut-off point for the corresponding significance level. The power from the semiparametric method under the *q*-value significance level of 0.1 is 0.052 for the 600 cases, and 0.054 for the 6000 cases, almost equal to Kulldorff's type I error level. Notice that higher *q*-value significance level means in favor of the alternative. If the *q*-value significance level is lower down to 0.05, the type I error of the semiparametric method reduced to 0.027 for both cases. It shows that if one wants to make the power comparison under exactly the same type I error level, say 0.05, the *q*-value significance level must be set in the interval between 0.05 to 0.1. In addition, the type I error of the semiparametric method with Bonferroni correction is the lowest, which is not a surprise, since Bonferroni correction is most conservative.

For scenarios No. 1 to 9, both figures show that the two methods work very well in detecting hot-spot clusters, but Kulldorff's method seems to be slightly more powerful. When the study area has a stronger pattern of clustering, such as containing more cluster regions, the power of both methods increases, which demonstrates the validity of the two methods. For instance, scenario No. 6 in Figure 3 has a total of 600 cases and two cluster regions where each cluster region contains 16 counties. The power of Kulldorff's method is 0.996, whereas the power of semiparametric method with *q*-value significance level of 0.1 and 0.05 obtain the power 0.996 and 0.993, respectively, which is quite close to the power of Kulldorff's. The semiparametric method with Bonferroni correction is 0.968, which is expected to be the lowest, but still is quite reasonable.

For scenarios No. 10 to 15, there is no clear cut to say Kulldorff's method or semiparametric method is better, and both methods do not achieve high power as in hot-spot detection. This is so because both Kulldorff's and the semiparametric methods are not designed to detect global clustering pattern. It is also shown that for both methods, the larger the *r *is, the lower is the detection power. This is reasonable, because larger *r *means larger range for the data to spread, hence more difficult to form a local cluster pattern.

#### Comparison for Ordinal Categorical Data

**Figure 5:**
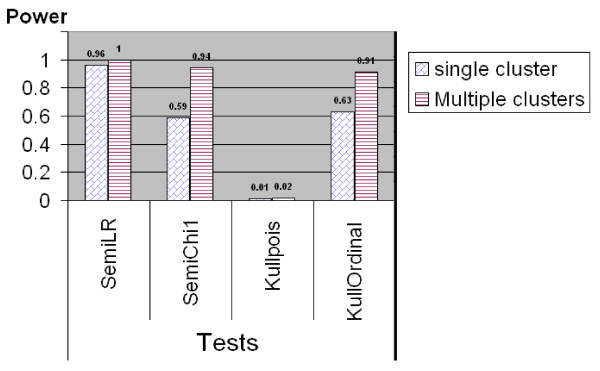
**Power comparison between Kulldorff's and the Semiparametric methods for ordinal categorical data generated from quantized normal II data, where the means are fixed at 13 but the variances are 4 and 8, between the cluster region and the rest of the area, respectively**.

**Figure 6:**
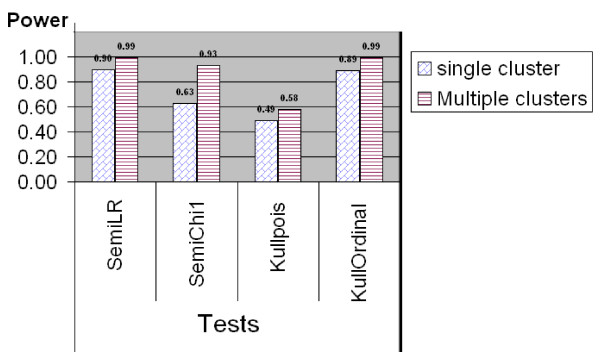
**Power comparison between Kulldorff's and the Semiparametric methods for ordinal categorical data generated from quantized normal III data, where means are 7.2 and 6, variance are 13 and 9, inside and outside the cluster region, respectively**.

**Figure 7:**
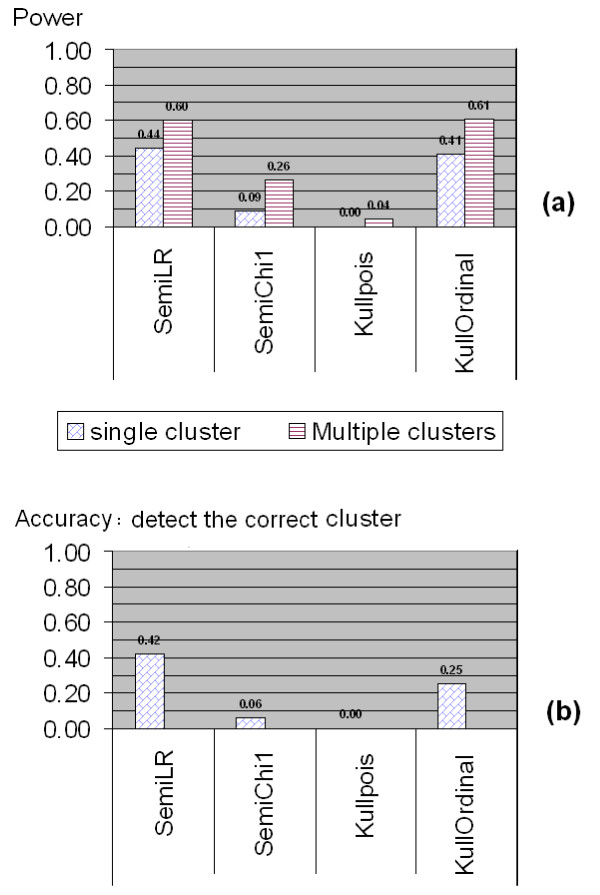
**Power comparison between Kulldorff's and the Semiparametric methods for ordinal categorical data with small difference. **The data are in quantized normal III small type with means 6.5 vs. 6 and variances 13 vs. 9, respectively.

The results of power comparison for non-binary ordinal categorical data using the simulated middle west and south US data set are shown in Figures 5, 6 and 7. Similar as in the power comparison for binary data, the radius of the scan window varies continuously from covering one county to covering no more than 50% of the whole study region. All the tests are two-sided tests. The significance level for Kulldorff's method is still 0.05, and the *q*-value significance level for the tests in the semiparametric method is set at 0.05 as well. This means the true type I error level of the semiparametric method might be lower than Kulldorff's as showed in the last subsection.

For ordinal categorical data which are generated from the quantized normal II type, Figure 5 shows that the semiparametric method with the likelihood ratio test has the highest power of detecting potential clusters among all the tests. The semiparametric method with the *χ*_1 _test works well but not as good as the likelihood ratio test. This is in line with the limited power study in Kedem and Wen (2007) [16] which showed the likelihood ratio test was the most powerful tests among three tests from the semiparametric density ratio model. Moreover, when the clustering pattern is stronger, for instance, the study region contains multiple clusters, the power of *χ*_1 _test increases to 0.94, which is almost close to the power of the likelihood ratio test. The power of Kulldorff's method with Poisson model is very low, because Poisson model is designed to detect the difference in the mean not in the variance. Kulldorff's method with ordinal model is comparable to the semiparametric method with the *χ*_1 _test in detecting potential clusters.

For ordinal categorical data which are generated from the quantized normal III type, Figure 6 shows that the semiparametric method performs in the same way as in the quantized normal II case. The semiparametric method with the likelihood ratio test still has the highest power in both single and multiple clusters situation. The power of all tests increases as the clustering pattern becomes stronger. Kulldorff's method with the ordinal model performs equally well as compared to semiparametric method. Kulldorff's method with Poisson model "works" but still much less powerful than the other tests. This is because for the quantized normal III data, Kulldorff's Poisson model can detect the changes in the mean while still ignoring changes in the variance.

The part (a) of Figure 7 shows the power results when the difference (in the mean) between the cluster and non-cluster regions is small. The data are still ordinal categorical data generated from the quantized normal III type. However, this time the difference is that the mean in the cluster region was set to be close to the the non-cluster region, while the variances inside and outside the cluster region were kept the same as in the previous case (refer to Table 2 for the simulation parameters). In this way, the clusters were more difficult to detect. Not surprisingly, the power for the semiparametric method with the likelihood ratio test and Kulldorff's method with the ordinal model continue achieving the highest power, although it is much lower than the power in the previous case where the differences inside and outside the cluster region were substantial. The power of the *χ*_1 _test of the semiparametric method also decreases due to the weaker cluster pattern, and Kulldorff's method with the Poisson model yields the lowest power almost 0. The multiple cluster case is similar to the single cluster case but with a relatively higher power. Interestingly, if we look at the accuracy, which means detecting the true cluster region correctly with its exact size, the most accurate method as shown in the part (b) of Figure 7 is the semiparametric method with the likelihood ratio test statistic, which achieves more than 50% higher accuracy rate than Kulldorff's method with the ordinal model.

### A Case Study — North Humberside childhood leukemia data set

**Figure 8:**
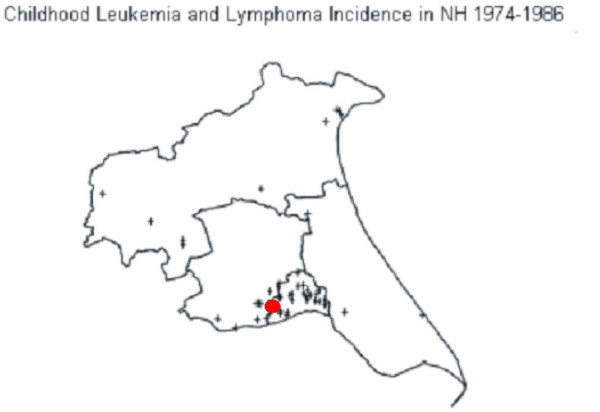
**Detected North Humberside childhood leukemia cluster denoted in red dot**.

The semiparametric method with Storey's *q*-value for adjusting the multiple-testing was again applied to the North Humberside childhood leukemia data set under the same conditions as used in Kedem and Wen (2007) [16]. As shown in Figure 8, both Kulldorff's method and the semiparametric method point to the same cluster as the primary cluster candidate, but but Kulldorff's method gave a highly insignificant *p*-value of 0.674, whereas the semiparametric method gave a significant *p*-value of 0.002 without adjusting for multiple testing and a close to boundary *q*-value of 0.073 adjusted by the Storey's approach. Thus, the results from the semiparametric method are more in line with the medical and environmental studies of Cuzick et al. (1990), Mckinney et al. (1991), and Colt and Blair (1998), which suggested that the North Humberside region had paternal exposure to solvents which were strongly associated with leukemia [39,41]. It seems the results from the semiparametric method that the located cluster candidate is a true cluster are more credible. More conclusive results may be obtained by increasing the sample size.

## Discussion and Conclusion

In this paper, We extended the semiparametric cluster detection method of Kedem and Wen (2007) [16] by incorporating Storey's *q*-value method, a type of FDR method, to overcome the multiple-testing problem raised by numerous scan windows of variable size. The results of the power study show that when detecting localized clusters, both the semiparametric and Kulldorff's method can achieve comparably good power. For binary population-case data, Kulldorff's method with Poisson model may have a slightly higher power than the semiparametric method with the tilt function *h*(*x*) = *x*. We may choose the tilt function as ***h***(*x*) = (*x, x*^2^)' for binary data as well although the *x*^2 ^term is not necessary. See Appendix 1 for a more detailed explanation. For non-binary data, such as ordinal categorical data, the semiparametric method with the tilt function ***h***(*x*) = (*x, x*^2^)' is slightly more powerful than Kulldorff's method with the ordinal model. If Kulldorff's method is applied with an inappropriate model, it may fail to detect any potential clusters as shown in the simulation study for non-binary data. We also find that in our semiparametric method the likelihood ratio test seems to have higher power than the *χ*_1 _test in detecting potential clusters. When the localized clustering pattern is strong, for instance, multiple cluster regions or the difference inside and outside the cluster region is large, both tests obtain good power. When the clustering pattern is weak, that is, the difference is not that large, the likelihood ratio test seems to be acuter, while the *χ*_1 _test could be insensitive to the undesired fluctuations. In addition, the Likelihood ratio test only can test "either high or low values", but *χ*_1 _test can be easily transformed into a one-sided test [16] for two-sample comparison and scalar tilt function. In practice, it is prudent to use both tests for potential clusters. Potentially, if one wants to borrow information from other sources besides the current study region, the *χ*_2_-test of the semiparametric method could be used.

In the power study for non-binary data, we only used the ordinal categorical data, which are integer data, to compare the power of the two methods. However, Kulldorff's method also provide exponential model to analyze the survival data, which are mostly assumed to be exponentially distributed, and a normal model to analyze continuous data. A future study is to compare the power of the semiparametric method and Kulldorff's method for continuous data. But we may expect the semiparametric method will still work well, since the semiparametric density ratio model was originated from continuous data. In addition, the semiparametric model may have a more consistent setup than Kulldorff's method. Kullorff's method needs to choose different models, namely different scan statistics, for different types of data, whereas the semiparametric method requires no complete distributional assumptions except for the tilt function ***h***(*x*). It could choose ***h***(*x*) = (*x, x*^2^)' for many types of data, no matter continuous or discrete.

If the underlying distribution is known exactly, semiparametric could choose the appropriate tilt function ***h***(*x*) to get the best performance. For instance, if the data are known from Bernoulli or Poisson distribution, we can use the tilt function *h*(*x*) = *x*; if the data are from normal, we can use ***h***(*x*) = (*x, x*^2^)'; if the data are from Gamma distribution, we can use ***h***(*x*) = (*x; *log *x*)'. A clue of how to choose a satisfactory ***h***(*x*) for a given situation can be derived from common exponential families. More examples can be found in Kedem and Wen (2007) or Kay and Little (1987) [16,42]. In addition, Fokianos et. al. (2001) [30] suggested that ***h***(*x*) could be approximated by a polynomial, B splines, or kernel estimation. If the underlying distribution is not known, there could be a problem of the misspecified tilt function. Fokianos and Kaimi has discussed that a misspecified ***h***(*x*) could decrease the power of the corresponding tests [43,44]. Yet, there are examples where very different choices of ***h***(*x*) could lead to similar test results. For instance, in an application to meteorological data in Kedem et al. (2004) [45] the choice of *h*(*x*) = *x *or *h*(*x*) = log *x *led to very similar results. It seems that for a non-homogeneous regional variance the choice of ***h***(*x*) = (*x, x*^2^)' suggested by the normal distribution is sensible.

The semiparametric density ratio model essentially tests the homogeneity or equidistribution of two or more samples, therefore, besides Kulldorff's circular scan window, the semiparametric method may also adapt to other shape of the scanning window or scanning schemes, such as the Kulldorff's elliptic window scan, irregular shape upper-level set scan, or flexible scan, etc. More precisely, in scanning for clusters, and regardless of the regular or irregular shape of the scanning window, as long as the window separates the whole study region into two samples, one inside the window and one outside the window, the semiparametric method can be applied. However, it also makes the semiparametric method ignore the information about the location of the cases except whether the case is inside or outside the current evaluated window. Thus the semiparametric method may not have good power for global type clustering as shown in scenarios 10 to 15 in Figure 3 and 4 where clustering occurs throughout the study region.

A limiting factor of this power study might be that in the power study for non-binary type data we only used 100 runs at one point for each power comparison. That is because it is tedious and time consuming to run SatScan software and document the results manually. In addition, the semiparametric method also takes longer time to numerically estimate the parameters *α *and *β *especially when the combined sample size is big, while for binary scan the MLEs of *α *and *β *have already been obtained in closed forms. However, although 100-run does not sound like a large number in simulation, the results are still meaningful. From Figures 5 and 6, it is already clear to show the semiparametric method achieves comparable good power as Kulldorff's method with the correct model achieves. If Kulldorff's method is applied with an inappropriate model, the power may decrease a lot. Figure 7 demonstrates that when the difference between the cluster and non-cluster region is small, the detecting power for both methods decrease, but the likelihood ratio test from the semiparametric method seems have better accuracy in detecting clusters.

A last note is about the *π*_0 _in Storey's *q*-value method. The critical part of the *q*-value method of controlling the false discovery rate is to give a good estimate of *π*_0_, the proportion of the true null hypotheses among all the tests. The current method we used in this study is based on the algorithm suggested by Storey et al. (2003) [35] and assume the distribution of *p*-value from each test is uniform over the (0,1) interval. However, Yang (2004) [46] pointed out that if the *p*-values were not uniformly distributed, the power of the *q*-value method may decrease. He suggested to compute a weighted average of *π*_0 _from the distribution of the raw *p*-values which are greater than a threshold (say 0.4). Thus it may give a better control and more robust estimate of *π*_0_.

## Appendix 1 — Simplified Semiparametric Test Statistics for Binary Data

If the data are 0-1 binary, such as cancer or not cancer, we can simplify the likelihood and obtain closed forms for the estimated parameters. Recall:

*N*_*G*_: The combined sample size for the whole study region.

*n*_*G*_: The number of cases in the whole study region.

*N*_*Z*_: The sample size within the scan window.

*n*_*G*_: The number of cases within the scan window.

*ti*: The *i*th observation from the combined sample in the whole study region, *i *= 1,..., *N*_*G*_.

*ρ*_1_: The relative sample size, which is equal to *N*_*Z*_/(*N*_*G *_- *N*_*Z*_).

*x*_*Zj*_: The *j*th observation within the scan window *Z*, *j *= 1,..., *N*_*Z*_.

For binary data, choose the tilt function *h*(*x*) = *x*. Then the profile log-likelihood with parameters *α*_1 _and *β*_1 _is(13)

The resulting maximum likelihood estimators are,(14)

If *β*_1 _= 0, it implies *α*_1 _= 0 and *e*^*α*1+*β*1 ^= 1, which means the relative rates inside and outside the scan window are equal.

By equation (7), then *χ*_1 _test statistic then can be simplified to(15)

Where  and  is as in equation (14).

By equation (9), then the likelihood ratio test statistic can be simplified to(16)

If the tilt function ***h***(*x*) is chosen as (*x,x*^2^)', the parameter *β*_11 _and *β*_12 _are associated with the *x *and *x*^2 ^term, respectively. Notice that for 0-1 binary data, *x *= *x*^2 ^in value, thus

the *x*^2 ^term is confounded with *x*, and *β*_12 _is not estimable. Therefore, tilt function *h*(*x*) = *x *with parameters *α*_1_, *β*_1 _are sufficient for binary case.

## Appendix 2 — Derivation of *S, V*

The matrices ***S, V ***are derived by repeated differentiation of the log-likelihood (eq. 17), which is the generalized version of equation (4) for *m *samples (*m *= *q *+ 1) with the *m*th sample being the reference sample.(17)

Recall that the asymptotic covariance matrix of the estimates in (5) is given by the product(18)

First define(19)

Then E[∇ *l*(*α*_1_,..., *α*_*q*_, ***β***_1_,..., ***β***_*q*_)]=**0**. To obtain the score second moments it is convenient to define *ρ*_*m *_≡ 1, *w*_*m*_(*t*) ≡ 1(20)

and,(21)

for *j, j' *= 1,..., *q*. Then, the entries in(24)

are(25)

The last term is 0 for *j *≠ *j' *and (*n*_*j*_/*n*)*Var *[***h***(∈_*j*1_)] for *j *= *j'*.

Next, as *n *→ *1*,(30)

where **S **is a *q*(1 + *p*) × *q*(1 + *p*) matrix with entries corresponding to *j, j' *= 1,..., *q*,(31)

## Competing interests

The authors declare that they have no competing interests.

## Authors' contributions

BK proposed the idea to apply the semiparametric density ration model to cluster detection. SW implemented the idea and developed the semiparametric cluster detection method. SW wrote the Splus code, conducted the power comparison, and wrote the manuscript. BK gave valuable comments to the manuscript. All authors have read and approved the final manuscript.
